# Vitamin B6 inhibits macrophage activation to prevent lipopolysaccharide‐induced acute pneumonia in mice

**DOI:** 10.1111/jcmm.14983

**Published:** 2020-01-22

**Authors:** Mei‐Rong Shan, Sheng‐Nan Zhou, Chang‐Ning Fu, Jia‐Wen Song, Xue‐Qing Wang, Wen‐Wu Bai, Peng Li, Ping Song, Mo‐Li Zhu, Zhi‐Min Ma, Zhan Liu, Jian Xu, Bo Dong, Chao Liu, Tao Guo, Cheng Zhang, Shuang‐Xi Wang

**Affiliations:** ^1^ The Key Laboratory of Cardiovascular Remodeling and Function Research The State and Shandong Province Joint Key Laboratory of Translational Cardiovascular Medicine Chinese Ministry of Education Chinese National Health Commission and Chinese Academy of Medical Sciences Qilu Hospital of Shandong University Jinan China; ^2^ Department of Traditional Chinese Medicine Qilu Hospital of Shandong University Jinan China; ^3^ College of Pharmacy Xinxiang Medical University Xinxiang China; ^4^ Department of Endocrinology Suzhou Science & Technology Town Hospital Suzhou China; ^5^ Department of Gastroenterology and Clinical Nutrition The First Affiliated Hospital of Hunan Normal University Changsha China; ^6^ Department of Cardiology Shandong Provincial Hospital Shandong University Jinan China; ^7^ Hubei Key Laboratory of Cardiovascular, Cerebrovascular, and Metabolic Disorders Hubei University of Science and Technology Xianning China

**Keywords:** AMP‐activated protein kinase, inflammation, lung, macrophage, vitamin B6

## Abstract

Macrophage activation participates in the pathogenesis of pulmonary inflammation. As a coenzyme, vitamin B6 (VitB6) is mainly involved in the metabolism of amino acids, nucleic acids, glycogen and lipids. We have previously reported that activation of AMP‐activated protein kinase (AMPK) produces anti‐inflammatory effects both in vitro and in vivo. Whether VitB6 via AMPK activation prevents pulmonary inflammation remains unknown. The model of acute pneumonia was induced by injecting mice with lipopolysaccharide (LPS). The inflammation was determined by measuring the levels of interleukin‐1 beta (IL‐1β), IL‐6 and tumour necrosis factor alpha (TNF‐α) using real time PCR, ELISA and immunohistochemistry. Exposure of cultured primary macrophages to VitB6 increased AMP‐activated protein kinase (AMPK) Thr172 phosphorylation in a time/dose‐dependent manner, which was inhibited by compound C. VitB6 downregulated the inflammatory gene expressions including IL‐1β, IL‐6 and TNF‐α in macrophages challenged with LPS. These effects of VitB6 were mirrored by AMPK activator 5‐aminoimidazole‐4‐carboxamide ribonucleoside (AICAR). However, VitB6 was unable to inhibit LPS‐induced macrophage activation if AMPK was in deficient through siRNA‐mediated approaches. Further, the anti‐inflammatory effects produced by VitB6 or AICAR in LPS‐treated macrophages were abolished in DOK3 gene knockout (*DOK3* ^−/−^) macrophages, but were enhanced in macrophages if DOK3 was overexpressed. In vivo studies indicated that administration of VitB6 remarkably inhibited LPS‐induced both systemic inflammation and acute pneumonia in wild‐type mice, but not in *DOK3* ^−/−^ mice. VitB6 prevents LPS‐induced acute pulmonary inflammation in mice via the inhibition of macrophage activation.

## 1 INTRODUCTION

Inflammatory diseases are mostly the result of uncontrolled inflammation, leading to damage and destruction of healthy tissues.^1^ Pneumonia, as one of lower respiratory illnesses mainly caused by pathogens, is associated with inflammatory stimulation from microorganisms, for instance, endotoxin. As a potent of endotoxin, lipopolysaccharide (LPS) is the main bioactive component of the cell wall of Gram‐negative bacterium and is critical to trigger the inflammatory response. Therefore, it is important to clarify the potential mechanism of inflammation and to develop some new effective strategies for treating pneumonia.

Multiple immune cells are involved in the process of lung inflammation including macrophages, lymphocytes and polymorphonuclear neutrophils. Activated macrophages release a variety of inflammatory factors such as reactive oxygen species, proteolytic enzymes and cytokines including interleukin‐1 beta (IL‐1β) and tumour necrosis factor alpha (TNF‐α), which promote severe damages in inflammation.^2^, ^3^ Thus, the inhibition of macrophage activation is regarded as an important therapeutic strategy in LPS‐induced pulmonary inflammatory disease.

Vitamin B6 (VitB6) includes pyridoxal, pyridoxine and pyridoxamine, which function as essential cofactors for enzymes involved in various metabolic activities, which include amino acid, fat and glucose metabolism.^4^ The phosphate ester derivative pyridoxal 5’‐phosphate (PLP) is the biologically active form of this vitamin and reflects long‐term body storage.^5^ Studies from others and us have shown that low plasma PLP concentrations are associated with increased risk of cardiovascular diseases^6^, ^7^ and supplementation of VitB6 lowers serum levels of IL‐6 and TNF‐α in patients with rheumatoid arthritis.^8^ VitB6 has also been demonstrated to suppress IL‐1β productions.^9^ Clinical trials have found that VitB6 alleviates Alzheimer's disease and Parkinson's disease.^10^, ^11^, ^12^ Whether VitB6 produces beneficial effects on pneumonia remains unknown.

The AMP‐activated protein kinase (AMPK) is a heterotrimeric protein composed of α, β and γ subunits.^13^ The α subunit imparts catalytic activity, while the β subunit contains a glycogen‐binding domain that also regulates the activity. The γ subunit forms the broad base of the protein and is required for AMP binding. We have previously reported that AMPK activation produces multiple protective effects in cardiovascular diseases by suppressing oxidative stress.^14^ Furthermore, hypoxia‐induced pulmonary arterial hypertension is accelerated in mice with AMPK ablation.^15^

The adaptor protein family downstream of kinase 1 (DOK1) to DOK3 is mainly found in immune cells, and they are closely related, with some functional redundancy.^16^ Mice deficient in DOK1 to DOK3 develop lung cancer and histiocytic sarcomas,^17^ revealing the functional importance of DOK proteins in regulating cellular responses in vivo. DOK1 and DOK2 function as negative regulators of TNF‐α production induced by lipopolysaccharide (LPS) in macrophages. DOK3 is associated with Toll‐like receptors (TLR) signalling stimulated by LPS and negatively regulates LPS responses and endotoxin tolerance in macrophages.^18^ DOK3 participates in the regulation of TLR signalling pathways by degradation or tyrosine phosphorylation.^19^

These mentioned studies support the notion that VitB6 may activate AMPK to inhibit LPS‐induced macrophage activation and to prevent acute pneumonia by activating DOK3. At present, the relationship between AMPK, macrophage activation, and DOK3 has not been fully defined. The aim of this study was to determine the effects and the molecular mechanisms of VitB6 in pulmonary inflammation. Our data suggest that the activation of AMPK pathway by VitB6 inhibits macrophage activation to prevent lung inflammation in vivo.

## 2 MATERIALS AND METHODS

A full description of materials and methods used, including reagents, animals, cell cultures, generations of adenovirus and infections, animal experimental protocol, LPS challenge in vitro and in vivo, real‐time PCR, ELISA and immunohistochemistry, HE staining, Western blot and statistical analysis can be found in the Online‐only Data Supplements.

### 2.1 Animals and protocols

Wild‐type (*WT*) B129 mice were generated by Beijing Wei Tong Li Hua Experimental Animal Technology Co Ltd. Gene knockout of DOK3 (*DOK3* ^−/−^) mice were kindly provided by Mary Beth Humphrey (Department of Microbiology and Immunology, University of Oklahoma Health Science Center). Mice were housed in temperature‐controlled cages with a 12‐hour light‐dark cycle and given free access to water and normal chow as we described previously.^20^ This study was carried out in strict accordance with the recommendations in the Guide for the Care and Use of Laboratory Animals of the National Institutes of Health. The animal protocol was reviewed and approved by the University of Shandong Animal Care and Use Committee.

### 2.2 In vivo LPS challenge

As described previously,^16^ three cohorts of age‐ and sex‐matched *WT* mice and *DOK3* ^−/−^ mice, male of 2 to 3 months of age, were pretreated with VitB6 (20 mg/kg) or saline for 6 hours and then injected with *S typhosa* LPS in phosphate‐buffered saline intraperitoneally at 0.5 mg/kg for 24 hours.

### 2.3 Determination of IL‐1β and TNF‐α

Gene expressions of IL‐1β and TNF‐α were determined by real time PCR. All PCR primers were generated by The Beijing Genomics Institute (BGI), and the sequences were shown in Table S1. The levels of secreted IL‐1β and TNF‐α in cultured medium and blood were assayed by ELISA. The protein levels of IL‐1β and TNF‐α in lung were measured by immunohistochemistry (IHC).

### 2.4 Statistical analysis

All quantitative results are expressed as mean ± SEM. One‐way ANOVA was used to compare multiple groups followed by Tukey's post hoc tests. Statistical analysis was conducted using IBM SPSS statistics 20.0 (IBM Corp), and *P* < .05 were considered as statistical significance.

## 3 RESULTS

### 3.1 VitB6 increases AMPK Thr172 phosphorylations in time‐ and dose‐dependent manners in macrophages

**Figure 1:**
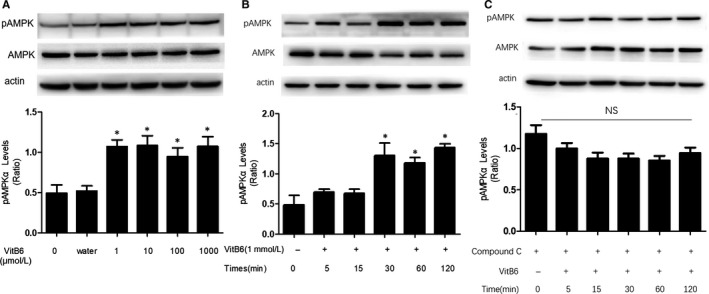
VitB6 increases AMPK phosphorylation in cultured macrophages. A, Cultured peritoneal macrophages were administrated with VitB6 in different concentrations (1, 10, 100, 1000 μmol/L) for 2 h. The levels of total and phosphorylated AMPK were detected by Western blotting method. N = 3 per group. *P < .05 vs Control (0 μmol/L). B, Cultured macrophages were treated with VitB6 (1 mmol/L) in different times. The levels of total and phosphorylated AMPK were detected by Western blotting method. The blot is a representative of five independent experiments. N = 5 per group. *P < .05 vs Control (0 min). C, Cultured macrophages were pretreated with Compound C (10 μmol/L) for 30 min followed by co‐incubation of VitB6 (1 mmol/L) for the indicated times. N = 3 per group. NS means no significance

Our earlier studies had solidly established that phosphorylation of AMPK at Thr172 correlates with AMPK activity in endothelial cells and vascular smooth muscle cells.^13^, ^14^, ^21^, ^22^, ^23^, ^24^, ^25^ Thus, we firstly proposed that VitB6 activates AMPK in macrophages by increasing AMPK Thr172 phosphorylations. Confluent peritoneal macrophages were treated with varying concentrations of VitB6 from 1 to 1000 μmol/L in culture medium for 2 hours. As shown in Figure 1A, VitB6 of 1000 μmol/L did not affect phosphorylation of AMPK at 15′ point. In contrast, VitB6 began to increase AMPK Thr172 phosphorylation at 30′. Increasing incubating time of VitB6 further enhanced AMPK phosphorylation. In Figure 1B, the phosphorylation of AMPK gradually increased beginning from 1 μmol/L after incubation with VitB6 for 2 hours and maintained the peak levels at 1000 μmol/L in cells. VitB6 treatment did not alter total levels of AMPK (Figure 1A,B). These data suggest that VitB6 is able to activate AMPK in macrophages.

### 3.2 VitB6‐induced AMPK phosphorylation is reversible in macrophages

To determine how VitB6 activates AMPK, we pretreated macrophages with compound C (10 μmol/L), a well‐recognized AMPK inhibitor,^13^ for 30 minutes, and then treated with VitB6. As shown in Figure 1C, we could see that the phosphorylation of AMPK increased by VitB6 was reversed in cells pretreated with compound C, compared to cells treated with vehicle. These data indicate that VitB6‐induced phosphorylation of AMPK is reversible.

### 3.3 VitB6 depresses the tyrosine phosphorylation of DOK3 and represses the association of DOK3 with AMPK

To explore the influence of VitB6 on the tyrosine phosphorylation of DOK3, we pretreated macrophages with VitB6 for 2 h and followed with LPS for 24 hours. As illustrated in Figure S1A and S2B, LPS increased the tyrosine phosphorylation of DOK3, compared with control group. However, it was decreased by VitB6. At the same time, to detect the association of DOK3 with AMPK α2, cells were preconditioned with VitB6 for 2 hours and then stimulated with LPS for 24 hours. LPS enhanced the association of DOK3 with AMPK but this association was weakened by VitB6, as shown in Figure S1C and S1D.

### 3.4 VitB6 inhibits LPS‐induced inflammatory gene expressions

**Figure 2:**
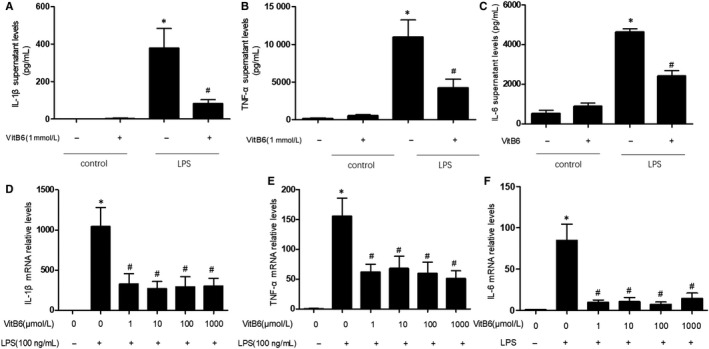
VitB6 inhibits LPS‐induced inflammation in cultured macrophages. Cultured macrophages were pretreated with vitamin B6 in different concentrations for two hours and then incubated with LPS (100 ng/mL) for 24 h. (A‐C) The protein levels of IL‐1β in A, TNF‐α in B and IL‐6 in C in culture medium were quantified by ELISA. (D‐F) The mRNA levels of IL‐1β in D, TNF‐α in E and IL‐6 in F were assayed by real‐time PCR. N = 3‐7 per group. *P < .05 vs Control. ^#^ *P* < .05 vs LPS

We have reported that AMPK activation produces the inhibitory effects on inflammation in multiple cells.^14^, ^24^ As a result, we would expect that VitB6 may suppress inflammation in culture macrophages. Inflammation was induced by LPS and assessed by measuring the levels of inflammatory cytokines, such as TNF‐α and IL‐1β, which are the inflammatory makers.^20^, ^26^ As depicted in, treatment of cultured macrophages with LPS for 24 hours dramatically induced the secretion of both TNF‐α, IL‐1β and IL‐6 as increased levels in cultured medium by ELISA (Figure 2A‐C), as well as increased gene expressional levels in total cells by real‐time PCR (Figure 2D‐F). However, pretreatment of cells with VitB6 dose‐dependently prevented the elevations of TNF‐α, IL‐1β and IL‐6 in culture medium and the upregulations of gene expressions (Figure 2A‐F). Together, it reveals that VitB6 is effective to inhibit LPS‐induced macrophage activation.

### 3.5 Pharmacological activation of AMPK by AICAR suppresses LPS‐induced inflammation in macrophages

To determine the role of AMPK in VitB6‐suppressed inflammation, we investigated whether the effects of VitB6 were duplicated by AICAR, which is an AMP analogue to activate AMPK by binding to AMPK grammar subunit.^21^ Liking VitB6, AICAR of 50 μmol/L remarkably reduced the mRNA levels of both TNF‐α and IL‐1β in LPS‐treated macrophages (Figure S2A and S2B), implying that VitB6 suppresses LPS‐induced inflammation, which is possibly mediated by AMPK activation.

### 3.6 AMPK is required for VitB6 to inhibit LPS‐induced inflammation in macrophages

**Figure 3:**
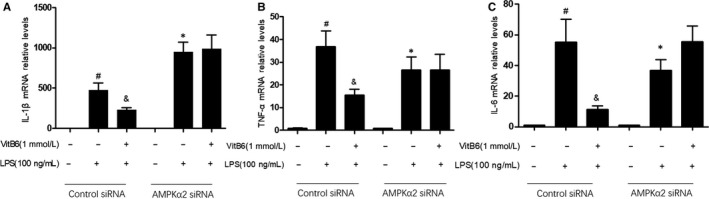
VitB6 via AMPK ablates LPS‐induced inflammation in cultured macrophages. Cultured macrophages were transfected with AMPKα2 siRNA or Control siRNA for 24 h followed by LPS (100 ng/mL) with or without VitB6 (1 mmol/L) pretreatment for 2 h. The mRNA levels of IL‐1β in A, TNF‐α in B and IL‐6 in C were measured by real‐time PCR. N = 3‐5 per group. ^#^ *P* < .05 vs Control siRNA alone; ^&^ *P* < .05 vs Control siRNA plus LPS; *P < .05 vs AMPKα2 siRNA alone

To exclude any potential off‐target effects of AICAR, we determined whether genetic inactivation of AMPK abolished the effects of VitB6‐inhibited inflammation in macrophages. To this end, cells were transfected for 24 hours with either negative control siRNA or AMPK siRNA and then exposed to VitB6. Enforced expression of AMPK siRNA, but not negative control siRNA, ablated VitB6‐induced downregulations of TNF‐α, IL‐1β and IL‐6 mRNA levels in total cell lysates (Figure 3A‐C). These data further confirm that AMPK activation is essential to the biological functions of VitB6 to inhibit LPS‐induced inflammation in macrophages.

### 3.7 DOK3 deficiency abolishes the inhibitory effects of VitB6 in LPS‐induced inflammation in macrophages

**Figure 4:**
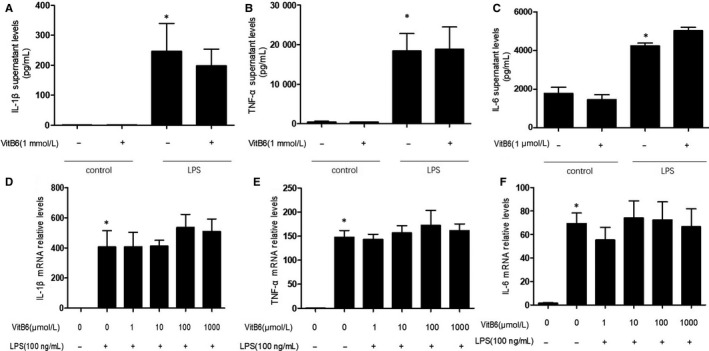
DOK3 deficiency abrogates the inhibitory effects of VitB6 on LPS‐induced inflammation in macrophages. Cultured *DOK3* ^−/−^ peritoneal macrophages were pretreated with VitB6 in different concentrations for 2 h and then incubated with LPS (100 ng/mL) for 24 h. (A‐C) The protein levels of IL‐1β in A, TNF‐α in B and IL‐6 in C in culture medium were quantified by ELISA. (D‐F) The mRNA levels of IL‐1β in D, TNF‐α in E and IL‐6 in F were assayed by real‐time PCR. N = 3‐7 per group. *P < .05 vs Control alone

As reported above, gene knockdown of AMPK by siRNA inhibited the effects of VitB6 on LPS‐induced inflammatory response. DOK3 is associated with LPS signaling and negatively regulates endotoxin tolerance in macrophages.^18^ Therefore, we thought DOK3 deficiency may produce similar effects of AMPK downregulation on VItB6’s function. We isolated primary peritoneal macrophages from *DOK3* ^−/−^ mice and then treated cells with VitB6 followed by LPS. As indicated in Figure 4A‐C, though LPS induced inflammation in cells deficient of DOK3, treatment of VitB6 did not lower the levels of TNF‐α, IL‐1β and IL‐6 in cultured medium. Further, VitB6 failed to inhibit the gene expressions of TNF‐α, IL‐1β and IL‐6 in primary peritoneal macrophages after LPS stimulation (Figure 4D‐F). In sum, DOK3 is required for VitB6 on the suppression of inflammation.

### 3.8 Vitamin B6 suppresses the expression of inflammatory factors induced by LPS through DOK3

To further uncover the roles of DOK3 in the function of VitB6 suppressing inflammation, we generated adenovirus expressing DOK3 cDNA and infected *DOK3* ^−/−^ macrophages with adenovirus for 24 hours followed with VitB6 and LPS. As illustrated in Figure S3A‐C, overexpression of DOK3 reversed the function of VitB6 to inhibit LPS‐induced inflammation by decreased the levels of TNF‐α, IL‐1β and IL‐6 in *DOK3* ^−/−^ cells, compared to *DOK3* ^−/−^ cells infected with adenovirus vector alone. These data indicate that VitB6‐suppressed inflammation is DOK3‐dependent.

### 3.9 DOK3 deficiency ablates the effects of AICAR in LPS‐induced inflammatory in primary peritoneal macrophages

**Figure 5:**
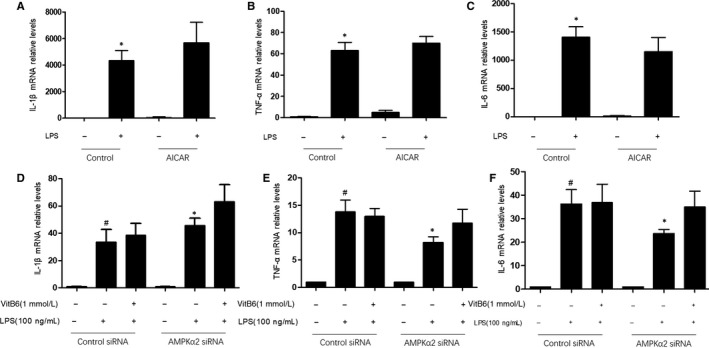
DOK3 mediates the functions of AMPK on LPS‐induced inflammation in macrophages. (A‐C) Cultured *DOK3* ^−/−^ macrophages were pretreated with AICAR (50 μmol/L) for 30 min followed by LPS (100 ng/mL) for 24 h. The mRNA levels of IL‐1β in A, TNF‐α in B and IL‐6 in C were confirmed by real‐time PCR. N = 3 per group. *P < .05 vs Control alone. (D‐F) Cultured *DOK3* ^−/−^ macrophages were transfected with AMPKα2 siRNA or Control siRNA for 24 h followed by LPS (100 ng/mL) with or without VitB6 (1 mmol/L) treatment for 2 h. The mRNA levels of IL‐1β in D, TNF‐α in E and IL‐6 in F were assayed by real‐time PCR. N = 4 per group. ^#^ *P* < .05 vs Control siRNA alone; *P < .05 vs AMPKα2 siRNA alone

Since we have identified the vital role of DOK3 in VitB6‐suppressed inflammation, we next determined whether DOK3 functions as a downstream of AMPK in this process. We pretreated *DOK3* ^−/−^ peritoneal macrophages with AICAR for 30 minutes to activate AMPK and then incubated cells with LPS for 24 hours. As shown in Figure 5A‐C, AICAR did not reduce the mRNA levels of TNF‐α, IL‐1β and IL‐6 in *DOK3* ^−/−^ macrophages, which were different to *WT* macrophages (Figure S2A‐C), suggesting that DOK3 is involved in the process of AMPK activation to inhibit inflammatory response.

### 3.10 DOK3 deficiency ablates the effects of AMPK siRNA in LPS‐induced inflammation in primary peritoneal macrophages

If DOK3 mediates the effects of AMPK activation to inhibit inflammation, we reasoned that DOK3 deficiency would balance the effects of AMPK gene knockdown on LPS‐induced inflammation. Thus, we transfected *DOK3* ^−/−^ peritoneal macrophages with siRNA and then treated with VitB6 or LPS. As represented in Figure 5D‐F, similar to LPS, AMPK siRNA induced inflammation in primary peritoneal macrophages. Furthermore, AMPK siRNA did not induce inflammation in *DOK3* ^−/−^ cells, demonstrating that DOK3 serves as a downstream mediator of AMPK contributing to VitB6‐induced the suppression of inflammation.

### 3.11 VitB6 prevents acute pneumonia in WT mice but not in *DOK3^‐/‐^* mice challenged with LPS

**Figure 6:**
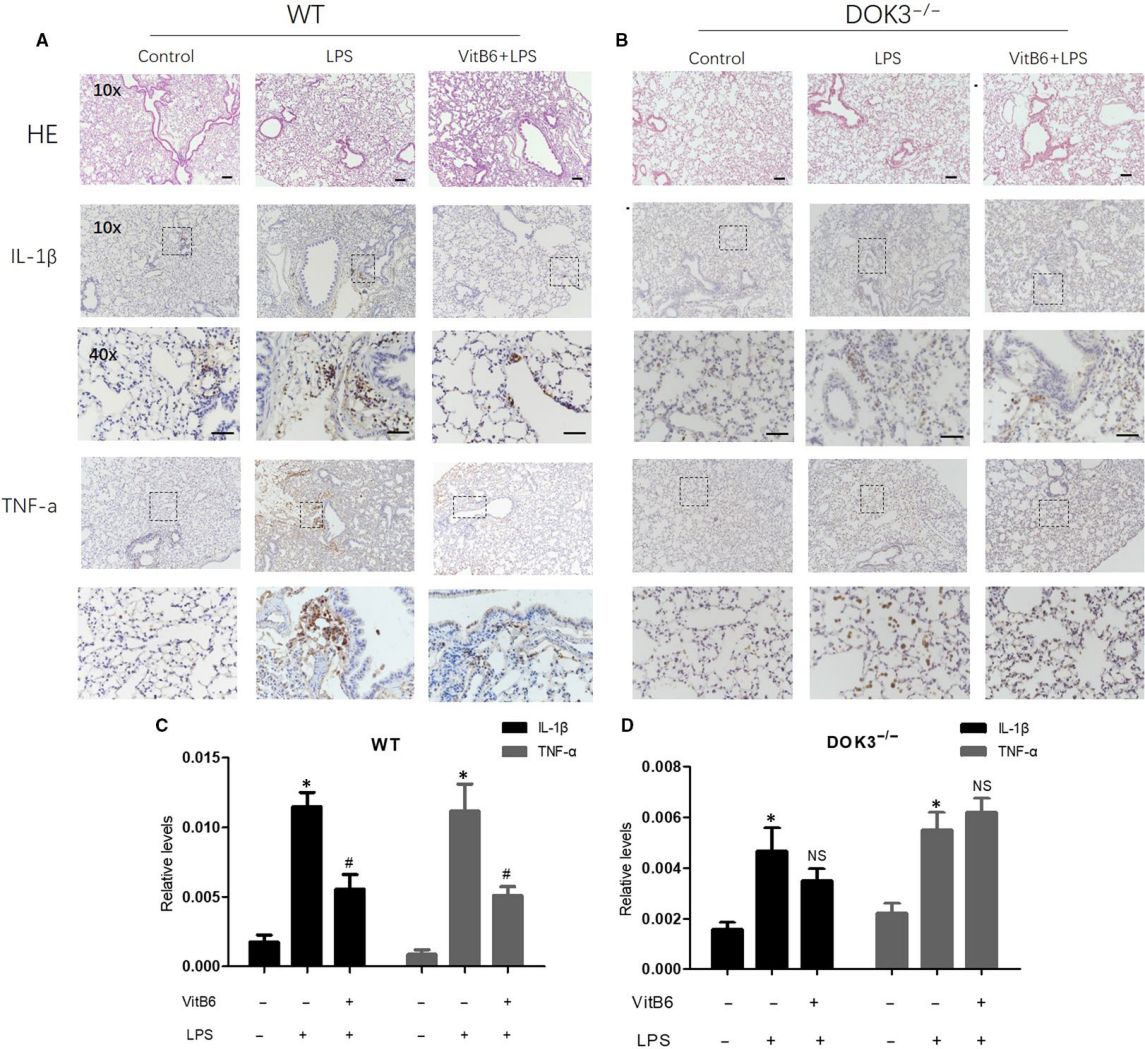
VitB6 represses LPS‐induced pulmonary inflammation in mice, which is DOK3 dependent. *WT* and *DOK3* ^−/−^ mice were pretreated with VitB6 (20 mg/kg) for 6 h followed by LPS (0.5 mg/kg) injection for 24 h. Lung tissue was fixed in paraformaldehyde for at least 48 h. (A and B) Lung tissue of both wild and *DOK3* ^−/−^ mice were subjected to perform histological morphology analysis by HE staining and IHC analysis of IL‐1β and TNF‐α. (C and D) Quantitative analysis of IL‐1β and TNF‐α of *WT* and *DOK3* ^−/−^ in C and D, respectively, was shown. N = 8‐12 per group. *P < .05 vs Control. ^#^ *P* < .05 vs LPS

Knowing that VitB6 activates AMPK‐DOK3 pathway to suppress inflammation in macrophages, we next detected the in vivo effects of VitB6 on LPS‐induced lung inflammation in mice. The model of lung inflammation was induced by injection of LPS in *WT* and *DOK3* ^−/−^ mice as described previously.^27^ Mice were challenged with an i.p. injection of VitB6 (20 mg/kg) followed by LPS (0.5 mg/kg) injection (Figure S4A). Twenty‐four hours later, the expressions of TNF‐α and IL‐1β in lung tissue were determined by IHC analysis. As shown in Figure 6A‐F, IHC analysis indicated that the levels of TNF‐α and IL‐1β were increased in lung tissue isolated from LPS‐injected *WT* mice and *DOK3* ^−/−^ mice. However, administration of VitB6 reduced the levels of TNF‐α and IL‐1β in lung isolated from LPS‐injected *WT* mice, but not in *DOK3* ^−/−^ mice. Besides, VitB6 increased AMPK phosphorylation in lung tissues isolated from both *WT* mice and *DOK3* ^−/−^ mice (Figure S4B,C). Taking these data together, it indicates that VitB6 is effective to inhibit LPS‐induced pulmonary inflammation in vivo through AMPK‐DOK3 signalling.

### 3.12 Gene knockout of DOK3 ablates the effects of VitB6 on LPS‐induced systemic inflammation in mice

The anti‐inflammatory effects of VitB6 were further confirmed by measuring serum levels of TNF‐α and IL‐1β in *WT* and *DOK3* ^−/−^ mice injected with LPS. As demonstrated in Figure S5A‐F, ELISA analysis demonstrated that LPS totally induced systemic inflammation as increased levels of TNF‐α and IL‐1β in blood isolated from *WT* and *DOK3* ^−/−^ mice. As expected, VitB6 significantly decreased the levels of TNF‐α and IL‐1β in blood isolated from LPS‐injected *WT* mice, but not in *DOK3* ^−/−^ mice. These data further support the viewpoint that VitB6 via the activation of AMPK‐DOK3 pathway functions as a reagent against acute pulmonary inflammation.

## 4 DISCUSSION

In the present study, we provided the evidence to determine that supplementation of VitB6 effectively prevents lung inflammation. We also showed that VitB6 via AMPK‐DOK3 pathway inhibits macrophage activation. In cultured cells, VitB6 increases AMPK phosphorylation to increase LPS‐induced inflammation. In mice, loss of DOK3 abolished the effects of VitB6 in suppression of lung inflammation. Thus, we conclude that AMPK‐DOK3 pathway is required for VitB6‐reduced inflammation to prevent pneumonia.

The major discovery of the present study is that VitB6 produces several beneficial effects to prevent inflammation in lung. Traditionally, VitB6 may treat depression, stroke, anaemia, nausea during pregnancy, clogged arteries, eye diseases, diabetes and inflammation associated with rheumatoid arthritis.^28^, ^29^, ^30^, ^31^ Recently, we have identified that VitB6 improves insulin resistance in *Apoe* ^−/−^ mice fed with high‐fat diet^32^ and prevents isocarbophos‐induced vascular dementia in rats.^33^ Here, we further demonstrated that VitB6 suppresses pulmonary inflammation by inhibiting macrophage activation, as reduced productions of TNF‐α and IL‐1β in vivo, consistent with other reports in patients with rheumatoid arthritis.^8^ Actually, many clinical trials have demonstrated that VitB6 alleviates Alzheimer's disease,^10^ Parkinson's disease^11^ and colorectal cancer,^12^ which are inflammation‐associated diseases.

Mechanistically, we uncovered that the AMPK‐DOK3 pathway contributes to the anti‐inflammatory effects of VitB6 by inhibiting macrophage activation. In general, VitB6, in the form of PLP, is the coenzyme of 5 enzymes in these metabolic pathways: cystathionine‐β‐synthase, cystathionine‐γ‐lyase, cytoplasmic and mitochondrial serine hydroxymethyltransferase and glycine decarboxylase in the mitochondria.^34^ In this way, VitB6 regulates the transsulfuration pathway, which contributes to homocysteine regulation and provides cysteine synthesis.^35^ However, in this study, we reported that AMPK‐DOK3 pathway mediates VitB6’s actions of anti‐inflammation. This notion is supported by the evidence as follows. First, we identified that VitB6 increases AMPK Thr172 phosphorylation in LPS‐treated macrophages. Second, loss function of AMPK by pharmacological inhibitor or gene silence abolishes the anti‐inflammation effects of VitB6. Third, gene knockout of DOK3 both in vitro and in vivo, liking AMPK downregulation, ablates VitB6’s anti‐inflammatory effects. Importantly, DOK3 deficiency bypasses the effects of VitB6, while DOK3 overexpression mimics the effects of AMPK activation by VitB6 or AICAR. All these data suggest that AMPK‐DOK3 signalling mediates VitB6’s functions as a suppressor of inflammation.

An issue needs to be discussed is that VitB6 has been reported to inhibit inflammation via suppressing NF‐κB activation and NLRP3‐mediated caspase‐1 activation because PLP is able to inhibit the phosphorylation of TAK1 and JNK induced by LPS as well as IKK‐IκBα pathway.^9^ However, our data reveal that VitB6 inhibits inflammation through AMPK Thr172 phosphorylation followed by DOK3 activation. In consistency, the inactivation of AMPK promotes a state of increased inflammatory responsiveness in murine macrophages, which is demonstrated by activating NF‐κB‐dependent gene expressions, as well as caspase‐1 activation and IL‐1β maturation.^36^ AICAR, as a well‐known AMPK activator,^21^ suppresses the releases of inflammatory cytokines induced by angiotensin II.^37^ Metformin, as an antidietetic drug by activating AMPK, inhibits inflammation induced by LPS in vascular cells.^23^, ^24^ In lung, suppressing AMPK aggravates LPS‐induced lung injury, hypoxia‐induced pulmonary arterial hypertension and endothelial barrier dysfunction.^24^ Considering these results, it is out of question that AMPK plays a crucial role in VitB6‐inhibited inflammation in macrophage.

Interestingly, DOK3 phosphorylation is usually mediated by tyrosine kinase. However, AMPK is serine or threonine kinase. In this study, we reported that AMPK activation by VitB6 or AICAR increased DOK3 tyrosine phosphorylation. We reasoned that AMPK may also phosphorylate some protein at tyrosine residue or AMPK activates some tyrosine kinases to phosphorylate DOK3. These issues need further investigations.

In summary, the present study we identified a novel action of VitB6 as an anti‐inflammatory reagent in response to LPS in lung and supplementation of VitB6 as a therapy against acute pneumonia. We also propose a role of AMPK‐DOK3 signalling in VitB6’s anti‐inflammatory effects (Figure S6). In perspective, AMPK or DOK3 activator may be a new drug to prevent pneumonia.

## CONFLICT OF INTEREST

None.

## AUTHOR’S CONTRIBUTIONS

MRS performed most experiments and wrote the manuscript. SNZ, CNF, JWS, XQW, WWB, PL, PS, MLZ, ZMM, ZL, JX, BD and CL partially performed some experiments. TG and CZ gave many critical suggestions to this project. SXW convinced the whole project and revised the manuscript.

## Data Availability

The datasets used and analysed during the current study are available from the corresponding author on reasonable request.
