# Supplementary material for: Vitamin B6 inhibits macrophage activation to prevent lipopolysaccharide‐induced acute pneumonia in mice
Source: J Cell Mol Med. 2020 Jan 22;24(5):3139–48. doi: 10.1111/jcmm.14983 (PMC7077594; doi:10.1111/jcmm.14983)
Supplement: Supplementary file 1 [file JCMM-24-3139-s001.pdf]

Online Supplements

Vitamin B6 inhibits macrophage activation to prevent lipopolysaccharide-induced acute pneumonia in mice

Mei-Rong Shan[1], Sheng-Nan Zhou[1], Chang-Ning Fu[1], Jia-Wen Song[1], Xue-Qing Wang[1], Wen-Wu Bai[1,2], Peng Li[3], Ping Song[3], Mo-Li Zhu[3], Zhi-Min Ma[4], Zhan Liu[5], Jian Xu[3], Bo Dong[6], Chao Liu[7], Tao Guo[1,*], Cheng Zhang[1,*], Shuang-Xi Wang[1,3,7,*]

[1]The Key Laboratory of Cardiovascular Remodeling and Function Research, Chinese Ministry of Education, Chinese National Health Commission and Chinese Academy of Medical Sciences, The State and Shandong Province Joint Key Laboratory of Translational Cardiovascular Medicine, Qilu Hospital of Shandong University, Jinan, Shandong, China; [2]Department of Traditional Chinese Medicine, Qilu Hospital of Shandong University, Jinan, Shandong, China; [3]College of Pharmacy, Xinxiang Medical University, Xinxiang, China; [4]Department of Endocrinology, Suzhou Science & Technology Town Hospital, Suzhou, China; [5]Department of Gastroenterology and Clinical Nutrition, The First Affiliated Hospital of Hunan Normal University, Changsha, China; [6]Department of Cardiology, Shandong Provincial Hospital, Shandong University, Jinan, China; [7]Hubei Key Laboratory of Cardiovascular, Cerebrovascular, and Metabolic Disorders, Hubei University of Science and Technology, Xianning, China

[*]Correspondences to Tao Guo, Cheng Zhang, and Shuang-Xi Wang, Email guotao@sdu.edu.cn, zhangc@sdu.edu.cn, and shuangxiwang@sdu.edu.cn

Short Title: Vitamin B6 prevents pneumonia

Materials and Methods

Reagents

Vitamin B6 (VitB6) was purchased from Sigma-Aldrich. 5-aminoimidazole-4-carboxamide ribonucleoside (AICAR) and compound C were brought from Calbiochem company. Antibodies against AMKP, pAMPKα-Thr172, DOK3, IL-1β, TNF-α, IL-6 and β-actin were purchased from Cell Signaling Company, Santa Cruz Biotechnology, or Abcam Company. Secondary antibodies were from Wuhan Boster Company. TIANamp Genomic DNA kit was purchased from TIANGEN BIOTECH (Beijing). AMPK siRNA was obtained from Shanghai Genepharma Company. The adenoviral vectors containing adenovirus DOK3 cDNA was purchased from Hanbio Biotechnology Co.Ltd. PimeScript^{TM}RT reagent Kit with gDNA Eraser and SYBR Green were from Takara Company. All ELISA kits were purchased from Research and Development (R&D) Company. All concentrations were expressed as the final molar concentration in the working buffer.

Animals and protocols

Wild type (*WT*) B129 mice were generated by Beijing Wei Tong Li Hua Experimental Animal Technology Co. LTD (Beijing, China). Gene knockout of adaptor protein downstream of kinase3 (*DOK3*$^{-/-}$) mice were kindly provided by Mary Beth Humphrey from University of Oklahoma Health Science Center. Mice were housed in temperature-controlled cages with a 12-hrs light-dark cycle and given free access to water and normal chow. This study was carried out in strict accordance with the recommendations in the Guide for the Care and Use of Laboratory Animals of the National Institutes of Health. The animal protocol was reviewed and approved by the University of Shandong Animal Care and Use Committee.

In vivo LPS challenge

As described previously[1], three cohorts of age- and sex-matched *WT* mice and *DOK3*$^{-/-}$ mice, male of 2 to 3 months of age, were pretreated with VitB6 (20mg/kg) for 6h and then injected with *S. typhosa* LPS in phosphate-buffered saline intraperitoneally at 0.5 mg/kg for 24h.

Cell cultures

As described previously[2], peritoneal macrophages were flushed from enterocoelia induced by Amylodextrine and maintained in completed medium. All cells were incubated at 37°C in a humidified atmosphere of 5% CO_2 and 95% air.

Transfection of siRNA to cells

Transfection of siRNA into macrophages was carried out according to Lipofectamine RNAimax Reagent protocol as described previously[3]. Shortly, the siRNAs powder was dissolved in DEPEC water to prepare a 20 μM stock solution. Cells were grown in 12 well plates and transfected with siRNA in opti-MEM (Gibicol), transfection medium, which contained liposomal transfection reagent (Lipofectamine RNAimax Reagent). 100 µl opti-MEM including 3 µl siRNA stock solution was gently mixed with 100 µl opti-MEM containing 3 µl Lipofectamine RNAimax. After 5-minute incubation at room temperature, the complexes were added to the 12-well plate with additional 300 µl opti-MEM, and incubated at 37°C, 5% CO_2 for 24 hours. The transfection medium was replaced with DMEM containing 10% fetal bovine serum, and macrophages were cultured for 24 hours.

Infection of adenovirus into cells

As described previously[4], peritoneal macrophages were incubated with adenovirus in DMEM containing 10% FBS for 24 hours and then replaced the medium for an additional 12 hours before treatment. Approximately 80% of cells were infected with overexpressed adenovirus.

Real-Time qPCR

Total RNA was extracted from treated cells with RNAfast2000 kit. The isolated mRNA was put on ice for the following steps, and then the total RNA concentrations were detected by employing a spectrophotometer, range of 260/280 ratio from 1.8 to 2.0. 1 µg of mRNA was applied for reverse transcription using PrimeCript^{TM} RT reagent kit with gDNA Eraser (TaKaRa, Japan) on the basis of the manufacturer's protocol. The mRNA levels of β-actin, IL-1β, TNF-α and IL-6 were detected by using SYBR Green technology (TaKaRa, Japan). The threshold cycle value (Ct) and the $2^{-\triangle\triangle CT}$ method were used for quantitative

calculation of relative mRNA levels as described previously[5]. All PCR primers were generated by The Beijing Genomics Institute (BGI) and the sequences were shown in Supplementary Table S1.

Supplementary Table S1.

Name	Forward	Reverse
β-actin	5´-CACTGTGCCCATCTACGA-3´	5´-GTAGTCTGTCAGGTCCCG-3´
IL-1β	5´-ACCTTCCAGGATGAGGACATGA-3´	5´-AACGTCACACACCAGCAGGTTA-3´
TNF-α	5´-AAGCAAGCAGCCAACCAG-3´	5´-TCTTCTGCCAGTTCCACG-3´
IL-6	5´-AGTTGCCTTCTTGGGACTGA-3´	5´-TCCACGATTTCCCAGAGAAC-3´

Enzyme-link immunosorbent assay

The supernatant of cultured macrophages and mouse serum were collected and stored in the -80°C. The concentrations of IL-1β and TNF-α were detected by using enzyme-link immunosorbent assay (ELISA) kits (Research and Development, USA), strictly following the instructions as described previously[6].

Western blot analysis

As described previously[7], various cell lysates and lung tissue homogenates were analyzed by Western blot. The concentrations of proteins were assayed by BCA protein assay kit. 20 μg protein samples were separated by SDS-PAGE and then transferred to polyvinylidene fluoride (PVDF) membranes, and the transferred time was determined by molecular weight. The membrane was blocked in 5% Bull Serum Albumin (BSA) for 2 hours at room temperature, then washed by PBS, 3 times and submerged in diluted primary antibodies (1:1000) for no less than 16h at 4°C, followed by secondary antibodies diluted in 1:5000. The final step was that bands attached with proteins were visualized by using the ECL reagent (Millipore Corp., MA, USA) to image. The levels of different proteins were detected by densitometry.

Immunohistochemistry (IHC)

As described previously[8], formaldehyde-fixed lung tissue paraffin sections were hydrated with dimethylbenzene and different concentrations of alcohol. Sections

were incubated with primary antibodies (IL-1β, TNF-α, CD68 and IL-6) overnight at 4°C, after antigen retrieval and blocking 5% BSA. Slides were washed with PBS and incubated with immunohistochemical secondary antibodies and then DAB chromogen was used to determine the levels of proteins in lung tissue; slides were stained with hematoxylin followed DAB chromogen. Finally, slides were sealed with natural gum mounting medium, observed and photographed under microscopy (Nikon). Ensured negative control was no staining and positive stained area was semi-quantitatively analyzed.

Statistical Analysis

All quantitative results are expressed as mean ± s.e.m. One-way ANOVA was used to compare multiple groups followed by Tukey *post-hoc* tests. Statistical analysis was conducted using IBM SPSS statistics 20.0 (IBM Corp., Armonk, NY, USA) and $P<0.05$ were considered as statistical significance.

References

1. **Chniguir A, Zioud F, Marzaioli V, El-Benna J, Bachoual R.** Syzygium aromaticum aqueous extract inhibits human neutrophils myeloperoxidase and protects mice from LPS-induced lung inflammation. *Pharm Biol*. 2019; 57: 56-64.
2. **Wang S, Zhang C, Zhang M, Liang B, Zhu H, Lee J, Viollet B, Xia L, Zhang Y, Zou MH.** Activation of AMP-activated protein kinase alpha2 by nicotine instigates formation of abdominal aortic aneurysms in mice in vivo. *Nature medicine*. 2012; 18: 902-10.
3. **Wang S, Xu J, Song P, Wu Y, Zhang J, Chul Choi H, Zou MH.** Acute inhibition of guanosine triphosphate cyclohydrolase 1 uncouples endothelial nitric oxide synthase and elevates blood pressure. *Hypertension*. 2008; 52: 484-90.
4. **Wang S, Zhang M, Liang B, Xu J, Xie Z, Liu C, Viollet B, Yan D, Zou MH.** AMPKalpha2 deletion causes aberrant expression and activation of NAD(P)H oxidase and consequent endothelial dysfunction in vivo: role of 26S proteasomes. *Circulation research*. 2010; 106: 1117-28.

5. **Wang J, Guo T, Peng QS, Yue SW, Wang SX.** Berberine via suppression of transient receptor potential vanilloid 4 channel improves vascular stiffness in mice. *Journal of cellular and molecular medicine*. 2015; 19: 2607-16.
6. **Li P, Yin YL, Guo T, Sun XY, Ma H, Zhu ML, Zhao FR, Xu P, Chen Y, Wan GR, Jiang F, Peng QS, Liu C, Liu LY, Wang SX.** Inhibition of Aberrant MicroRNA-133a Expression in Endothelial Cells by Statin Prevents Endothelial Dysfunction by Targeting GTP Cyclohydrolase 1 in Vivo. *Circulation*. 2016; 134: 1752-65.
7. **Wang S, Xu J, Song P, Viollet B, Zou MH.** In vivo activation of AMP-activated protein kinase attenuates diabetes-enhanced degradation of GTP cyclohydrolase I. *Diabetes*. 2009; 58: 1893-901.
8. **Yang XH, Li P, Yin YL, Tu JH, Dai W, Liu LY, Wang SX.** Rosiglitazone via PPARgamma-dependent suppression of oxidative stress attenuates endothelial dysfunction in rats fed homocysteine thiolactone. *Journal of cellular and molecular medicine*. 2015; 19: 826-35.

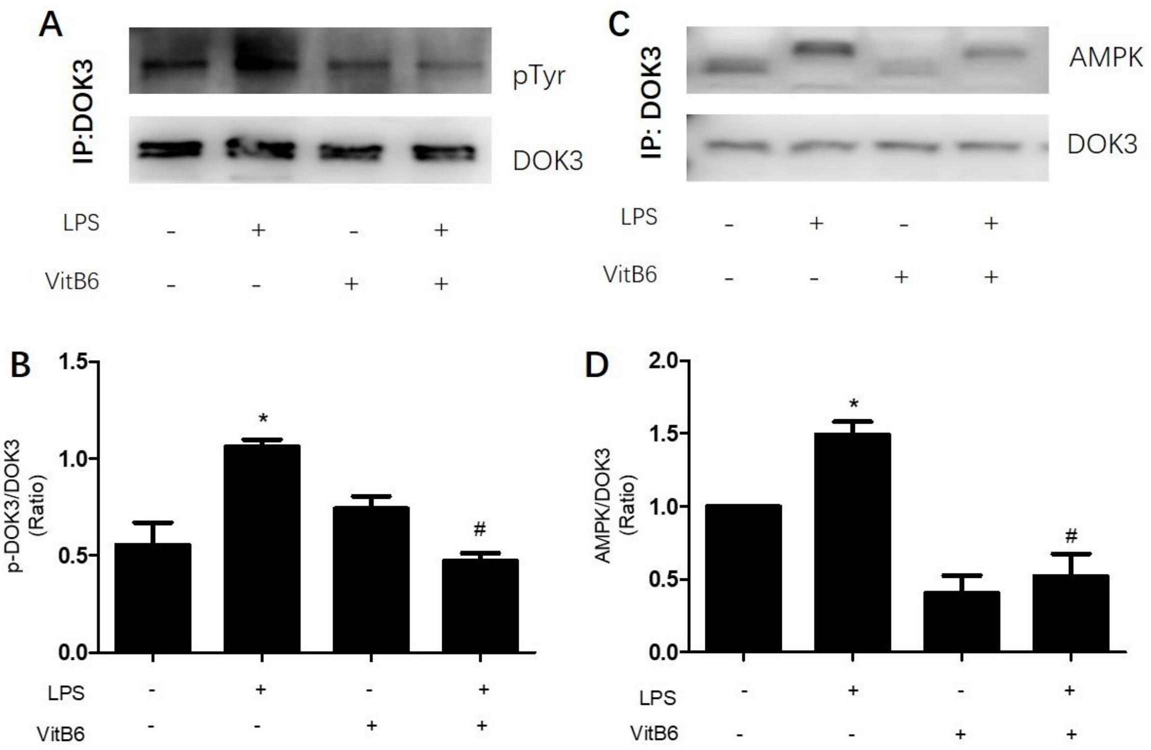

Figure S1. VitB6 inhibits tyrosine phosphorylation of DOK3 and represses the association of DOK3 with AMPK α2 in LPS-treated macrophages. Cultured macrophages were pretreated with vitamin B6 (1 mM ,2 hours) and followed by co-incubation LPS (100ng/ml) for 24 hours. (**A** and **B**) Total cell lysates were subjected to immunoprecipitation with anti-DOK3 antibody and analyzed by Western Blotting for pTyr and DOK3. (**C** and **D**) Cell lysates were subjected to immunoprecipitation with anti-DOK3 antibody and analyzed by Western Blotting for AMPK and DOK3.The blot is a representative of three independent experiments. N = 3 per group. *P<0.05 *vs*. Control. #P<0.05 *vs*. LPS.

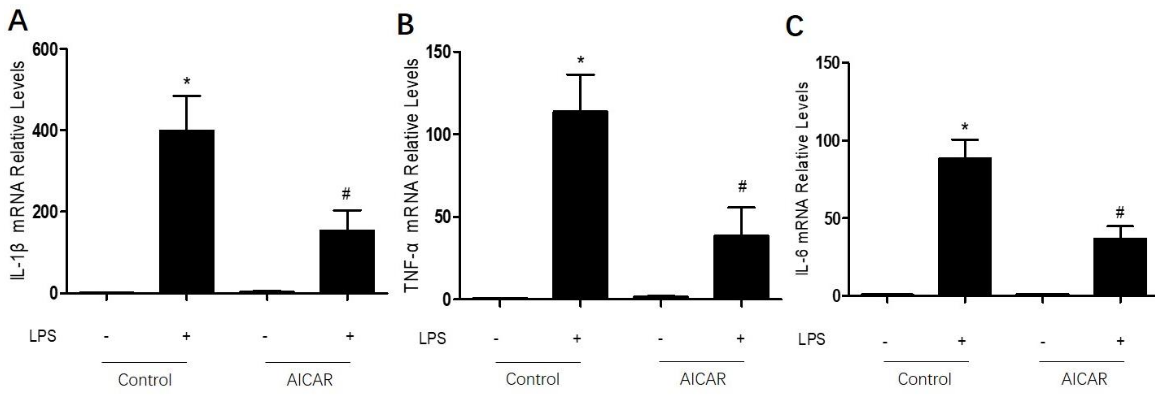

Figure S2. Pharmacological activation of AMPK suppresses LPS-induced inflammation in macrophages. Cultured macrophages were pretreated with AICAR (50 μM) for 30 min followed by LPS (100 ng/ml) for 24 hours. The mRNA levels of IL-1β in **A**, TNF-α in **B**, and IL-6 in **C** were assayed by real-time PCR. N = 3-7 per group. **P*<0.05 *vs*. Control. #*P*<0.05 *vs*. LPS.

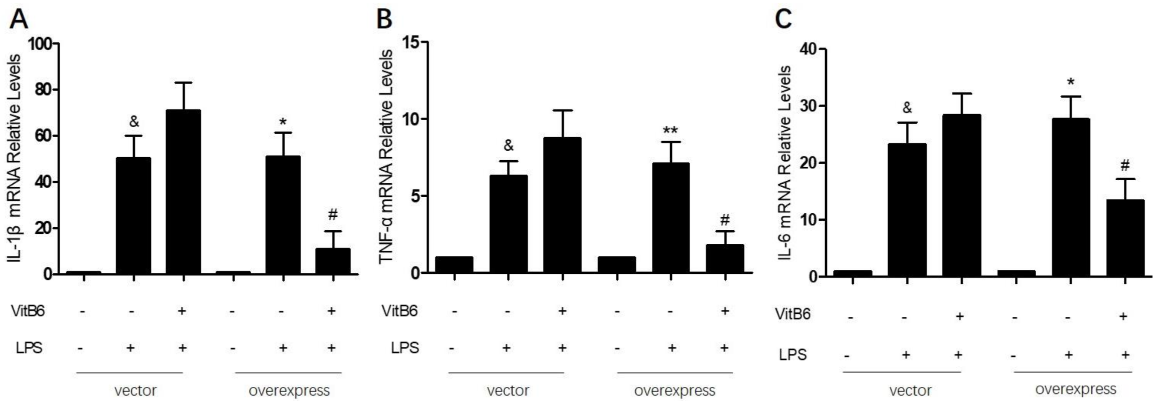

Figure S3. DOK3 overexpression enhances the effects of VitB6 on suppressing LPS-induced inflammation *in vitro*. Cultured *DOK3*$^{-/-}$ macrophages were infected with adenovirus expression DOK3 for 24 hours and followed with VitB6 (1 mM) for 2 hours and LPS (100 ng/ml) for 24 hours. The mRNA levels of IL-1β in **A**, TNF-α in **B**, and IL-6 in **C** were assayed by real-time PCR N = 3 per group. $^{\&}P<0.05$ *vs*. Vector alone; $^{*}P<0.05$ *vs*. Overexpress alone; $^{\#}P<0.05$ *vs*. Overexpress plus LPS.

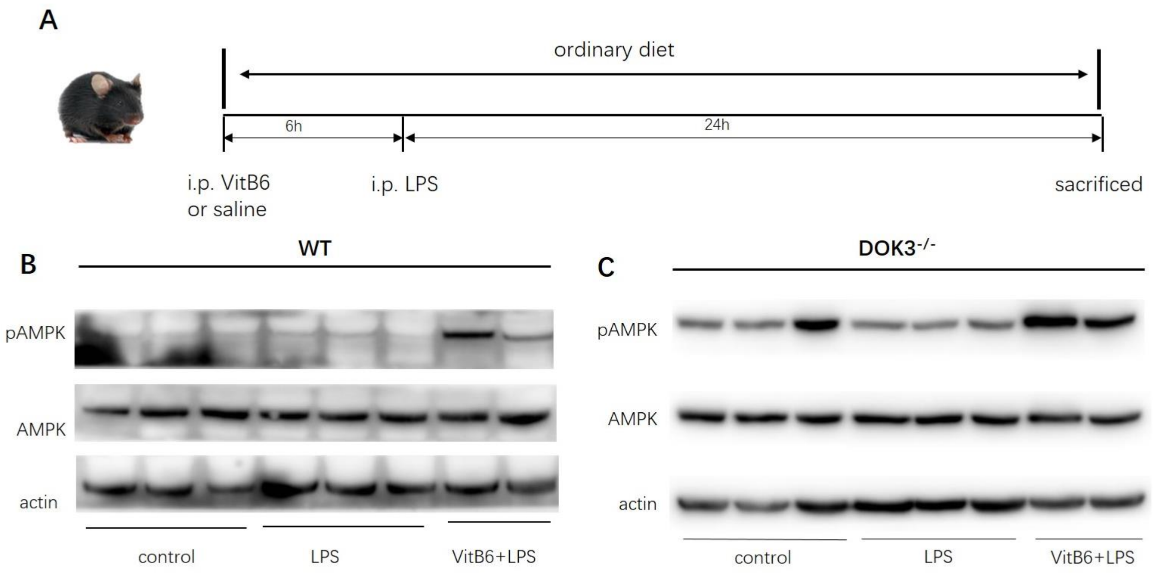

Figure S4. The animal experimental protocol and the *in vivo* efficacy of VitB6 on AMPK phosphorylation. (**A**) The protocol of animal experiments in mice. *In vivo*, (**B** and **C**) the effects of VitB6 on AMPK phosphorylation were assayed by western blot in WT and DOK3$^{-/-}$ mice lung tissue.

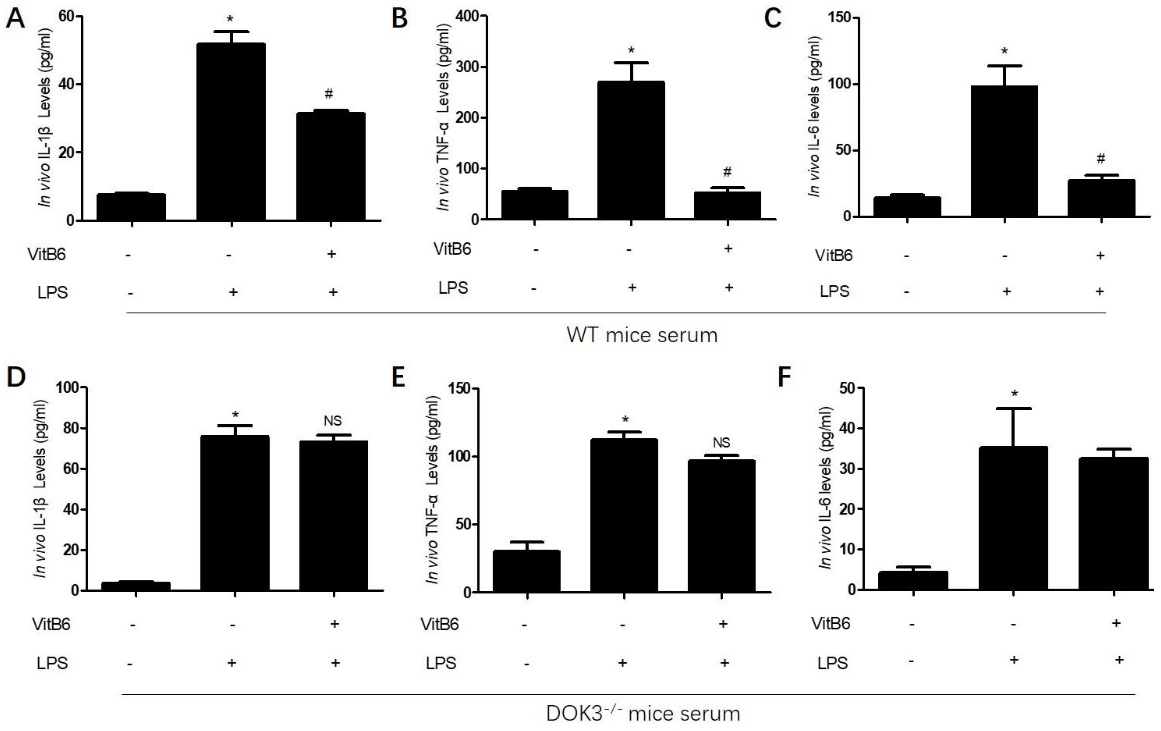

Figure S5. VitB6 represses LPS-induced systemic inflammation in mice, which is DOK3 dependent. *WT* and *DOK3*$^{-/-}$mice were pretreated with VitB6 (20 mg/kg) for 6 hours followed by LPS (0.5 mg/kg) injection for 24 hours. Serum was collected to measure cytokines by ELISA. Serum levels of IL-1β in **A**, TNF-α in **B**, and IL-6 in **C** in *WT* mice were shown. Serum levels of IL-1β in **D**, TNF-α in **E**, and IL-6 in **F** in *DOK3*$^{-/-}$ mice were shown. N = 4-7 per group. *P<0.05 *vs*. Control. $^{\#}P$<0.05 *vs*. LPS.

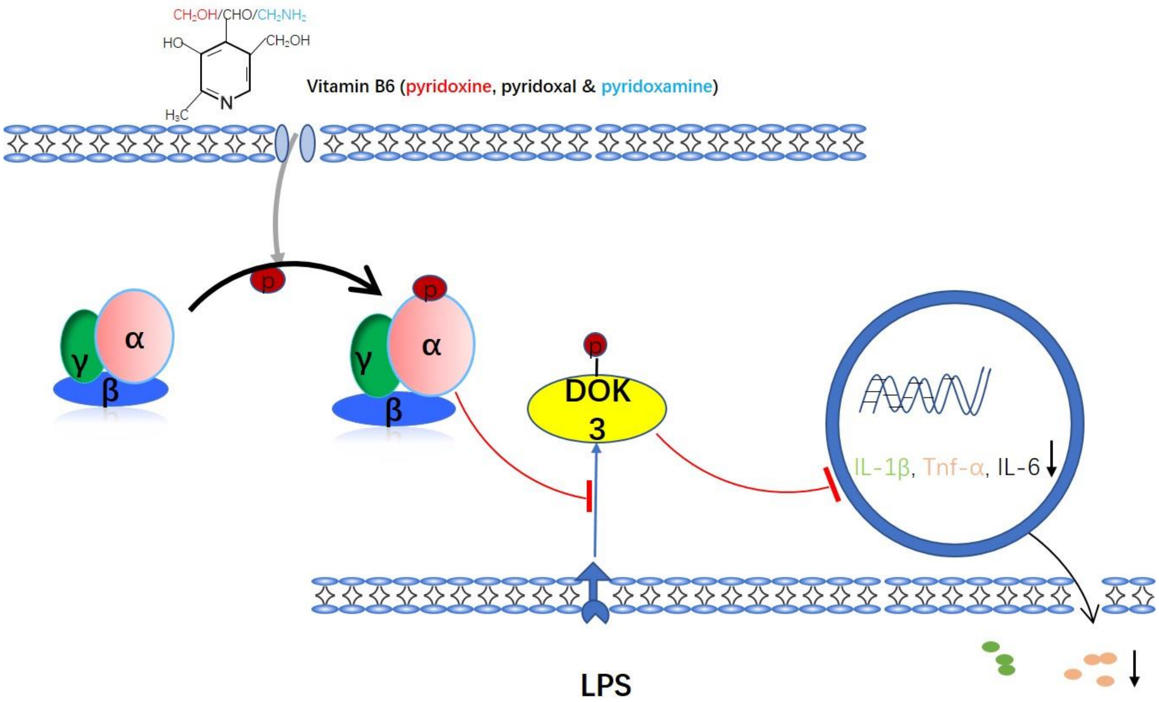

Figure S6. Proposed mechanisms by which VitB6 prevents inflammation. In macrophage, AMPK phosphorylation increases DOK3 activation, leading to the determination of LPS signaling. In this way, VitB6 produces the suppressive effects on the development of inflammation in lung through suppression of macrophage activation.
